# Bloodstream Infections in a COVID-19 Non-ICU Department: Microbial Epidemiology, Resistance Profiles and Comparative Analysis of Risk Factors and Patients’ Outcome

**DOI:** 10.3390/microorganisms10071314

**Published:** 2022-06-29

**Authors:** Efthymia Giannitsioti, Christina Louka, Vasiliki Mamali, Elisavet Kousouli, Lemonia Velentza, Vaia Papadouli, Georgios Loizos, Panagiotis Mavroudis, Georgios Kranidiotis, Nektaria Rekleiti, Alexandra Stamati, Ioannis Speggos, Ioannis Daniil, Panagiotis Kouvatsos, Chrysanthi Sidiropoulou, Garifallia Linardaki, Styliani Gerakari, Georgios Chrysos, Katina Themeli-Digalaki, Olympia Zarkotou

**Affiliations:** 1 COVID-19 Department, Tzaneio General Hospital, 18536 Piraeus, Greece; lemvele@gmail.com (L.V.); loizos.georgios@gmail.com (G.L.); panos.mavroudis@gmail.com (P.M.); gekranid@hotmail.com (G.K.); asgns95@gmail.com (A.S.); jspeggos@yahoo.gr (I.S.); panoskouvatsos1@hotmail.com (P.K.); x_sidiropoulou@hotmail.com (C.S.); garrylin11@yahoo.gr (G.L.); sgerakari76@gmail.com (S.G.); gchrysos@gmail.com (G.C.); 2 Fourth Department of Internal Medicine, NKUA, ATTIKON University General Hospital, 12462 Athens, Greece; 3 Department of Microbiology, Tzaneio General Hospital, 18536 Piraeus, Greece; cslouka@gmail.com (C.L.); mamalivasiliki@outlook.com.gr (V.M.); papadouli51@yahoo.gr (V.P.); nekrek@gmail.com (N.R.); drdaniel.gr@gmail.com (I.D.); kdigalaki@gmail.com (K.T.-D.); olyzar@hotmail.com (O.Z.); 4 Infection Control Action Team, Tzaneio General Hospital, 18536 Piraeus, Greece; elisavetkousouli@yahoo.gr; 5 Emergency Department, Tzaneio General Hospital, 18536 Piraeus, Greece; 6 Second Department of Internal Medicine, Tzaneio General Hospital, 18536 Piraeus, Greece

**Keywords:** COVID-19, SARS-CoV-2 pneumonia, bloodstream infections, bacteremia, fungemia, catheter-related bloodstream infection, antimicrobial resistance, multidrug resistant organisms

## Abstract

Background: Bloodstream infections (BSI) caused by highly resistant pathogens in non-ICU COVID-19 departments pose important challenges. Methods: We performed a comparative analysis of incidence and microbial epidemiology of BSI in COVID-19 vs. non-COVID-19, non-ICU departments between 1 September 2020-31 October 2021. Risk factors for BSI and its impact on outcome were evaluated by a case-control study which included COVID-19 patients with/without BSI. Results: Forty out of 1985 COVID-19 patients developed BSI. The mean monthly incidence/100 admissions was 2.015 in COVID-19 and 1.742 in non-COVID-19 departments. *Enterococcus* and *Candida* isolates predominated in the COVID-19 group (*p* < 0.001 and *p* = 0.018, respectively). All *Acinetobacter baumannii* isolates were carbapenem-resistant (CR). In the COVID-19 group, 33.3% of *Klebsiella pneumoniae* was CR, 50% of *Escherichia coli* produced ESBL and 19% of *Enterococcus* spp. were VRE vs. 74.5%, 26.1% and 8.8% in the non-COVID-19 group, respectively. BSI was associated with prior hospitalization (*p* = 0.003), >2 comorbidities (*p* < 0.001), central venous catheter (*p* = 0.015), severe SARS-CoV-2 pneumonia and lack of COVID-19 vaccination (*p* < 0.001). In the multivariate regression model also including age and multiple comorbidities, only BSI was significantly associated with adverse in-hospital outcome [OR (CI95%): 21.47 (3.86–119.21), *p* < 0.001]. Conclusions: BSI complicates unvaccinated patients with severe SARS-CoV-2 pneumonia and increases mortality. BSI pathogens and resistance profiles differ among COVID-19/non-COVID-19 departments, suggesting various routes of pathogen acquisition.

## 1. Introduction

The COVID-19 pandemic emerged as the most serious public health threat worldwide since the influenza pandemic of the last century. Millions of cases of infection and a subsequent proportion of attributed deaths were caused by this novel disease, which has seriously affected global healthcare systems along with social, administrative and economic life [1,2]. The causative virus, SARS-CoV-2, a novel coronavirus belonging to the subfamily of Coronavirinae in the family Coronaviridae, causes severe pneumonia often requiring hospital care [1]. Moreover, COVID-19 critical illness led to an increase in intensive care unit (ICU) admissions and a devastating rate of mortality [3]. Severe SARS-CoV-2 pneumonia leads to prolonged hospitalization and can be complicated by secondary bacterial and fungal infections with an increased negative impact on the patients’ outcome [4,5]. Co-infections are much more frequent in critically ill patients in the ICU (31.5%) than in hospitalized patients in standard medical COVID-19 wards (9%) [6]. Rates of bacterial co-infection are low at admission (3.5%), rising to more than 15% during hospitalization; overall, 7% of hospitalized patients will develop bacterial superinfection [4,7,8]. Both community-acquired and nosocomial bacterial co-infections were caused by Gram-negative bacteria such as *Pseudomonas aeruginosa* and *Escherichia coli*, while *Acinetobater baumannii* and *Klebsiella pneumoniae* were linked to a higher need for invasive mechanical ventilation (IMV) and increased in-hospital mortality [4,7]. Moreover, a high risk for secondary fungal infections among COVID-19 patients has been described [9]. Bloodstream infections are a significant subset of infections following acute SARS-CoV-2 pneumonia and a driver for sepsis and in-hospital mortality [10,11]. Current data suggest that diagnosis and subsequent prompt treatment of BSI might be delayed due to a focalized effort for controlling the primary infection (SARS-CoV-2 pneumonia) [12]. Moreover, nosocomial BSI is often caused by difficult-to-treat multidrug-resistant organisms (MDRO), especially in countries with high prevalence of antimicrobial resistance, posing additional therapeutic concerns [10,13].

The impact of BSI in patients hospitalized for COVID-19 has been studied through national or local cohorts; however, most studies were performed in critically ill patients in the ICU who present a higher mortality from a BSI event [3,8,10,13,14]. Clinical characteristics and outcome of patients with BSI and COVID-19 in medical wards might differ from patients hospitalized in the ICUs, and they are not fully investigated. For this purpose, we conducted a case-control study on risk factors for BSI and on the patients’ clinical outcome in COVID-19 medical wards, outside the ICU department. The incidence of BSI, the microbial epidemiology and antimicrobial resistance patterns were also compared between COVID-19 and non-COVID-19, non-ICU departments.

## 2. Materials and Methods

### 2.1. Study Protocol

We retrospectively analyzed prospectively collected data on the incidence, clinical characteristics and outcome of BSI in patients hospitalized for COVID-19 in the medical, non-ICU department of our tertiary-care hospital from 1 September 2020–31 October 2021. The incidence of BSI per 100 admissions, BSI pathogens and susceptibility profiles were also assessed in COVID-19 vs. non-COVID-19 departments of our hospital. Microbiological and clinical data from the ICU were not included in the study. 

Potential risk factors for BSI and its impact on mortality were evaluated through a case-control study. Patients with BSI were matched for age and gender at a 1:1 ratio with a randomly selected control group of patients without BSI hospitalized for COVID-19 pneumonia during the study period. Analysis of factors associated with adverse outcome defined as IMV and/or death was performed. Demographics, comorbidities, prior recent hospitalization or residency to a healthcare setting or nursing home, vaccination against SARS-CoV-2, the placement of a central venous catheter (CVC) during hospitalization and the length of hospital stay were recorded. Time from patients’ admission to the onset of BSI (date of collecting the first positive blood culture) was also assessed. Anonymized patients’ data were analyzed. 

### 2.2. Definitions

A BSI episode was documented by at least one positive blood culture and consistent clinical presentation. Common skin contaminants (such as coagulase-negative staphylococci (CoNs), *Micrococcus* spp., viridans group streptococci, *Propionibacterium* spp., *Corynebacterium* spp. and *Bacillus* spp.) were excluded unless they were recovered from at least two blood culture sets. Catheter-related BSI (CRBSI) was detected by catheter tip culture and/or differential time to positivity according to the CRBSI definitions by IDSA guidelines [15]. Multiple positive blood cultures for the same organism in the same patient were considered as one BSI episode in the present analysis. BSI episodes were categorized as community-acquired or hospital-acquired, depending on the time of blood sampling, within or after 48 h from admission.

Diagnosis of SARS-CoV-2 pneumonia relied upon clinical and radiological characteristics of the disease on lung X-ray or CT scan along with a positive nasopharyngeal swab test for SARS-CoV-2 (RT-PCR) according to current guidelines [16]. Severe SARS-CoV-2 pneumonia was classified according to the WHO clinical progression scale of the disease [17]. All hospitalized patients received remdesivir. Dexamethasone was administered only in patients with severe pneumonia.

In-hospital outcome was defined as “cure” when the patient was discharged at home or any other permanent hosting place and “non-cure” if the patient had either died or been intubated and transferred to the ICU.

### 2.3. Microbiology

Blood cultures were incubated using the BD BACTEC™ FX Automated Blood Culture System (Becton and Dickinson, Franklin Lakes, NJ, USA). Identification and susceptibility testing of blood isolates were performed by the Vitek 2 automated system (bioMerieux, Marcy l’ Etoile, France). MIC values for colistin were determined by broth microdilution (MIC-Strip Colistin, Merlin Diagnostika GmbH, Bornheim, Germany). EUCAST breakpoints were applied for interpretation of MIC values [18]. Phenotypic detection of carbapenemases and differentiation of carbapenemase type among Enterobacterales and *P. aeruginosa* isolates was performed by immunochromatography (NG-Test^®^ CARBA 5, NG Biotech, Guipry, France).

Gram-negative multidrug resistant (MDR) pathogens were defined according to Magiorakos et al. criteria [19]. MDR Gram-positive bacteria (GPB) included methicillin-resistant *Staphylococcus aureus* (MRSA) and vancomycin-resistant *Enterococcocus* spp. (VRE). 

### 2.4. Statistical Analysis 

Pearson chi square and Fischer exact tests were applied for categorical and the Mann–Whitney test for continuous variables in comparisons between BSI and non-BSI COVID-19 groups. Factors of in-hospital adverse outcome (“non-cured patients”) were identified via univariate and multivariate regression analysis. The primary dependent variable was outcome (cure vs. non-cure). The primary independent variable was BSI (either bacteremia or fungemia). Fisher’s exact test along with the odds ratio (OR) and 95% confidence interval (CI) were applied. The level of significance was set at 0.05. Statistical analyses were performed with SPSS version 22.0.

## 3. Results

### 3.1. Microbial Epidemiology 

During the study period, a total of 470 blood culture (BC) sets were obtained from 321 non-ICU hospitalized patients with COVID-19; 102 BC sets were detected as positive (26.9%). True pathogens were recovered from 49 BC sets. Contamination rate was 52%. A total of 45 BSI episodes in 40 patients were identified among 1985 non-ICU COVID-19 hospitalized patients (2%). Most patients were female, (*n* = 24, 60%) and the median age was 73.5 years [IQR (25–75): 64.5–85.25 years]. Multiple (more than 2) comorbidities, were found in 31 patients (77.5%) while 11 patients (27.5%) were either transferred from a nursing care center or had recent prior hospitalizations. The mean (±SD) time of hospitalization from admission to the onset of the BSI episode was 11 ± 7.84 days. All but 3 BSI cases fulfilled the definition of nosocomial infection. A CVC was inserted in 18 patients (90% into the femoral vein); CRBSI was documented in 4 patients (22.2%). Overall adverse outcome (in-hospital mortality or IMV) was noted in 60% of BSI cases. Sixteen patients required IMV; 14 of them died. Eight non-ventilated patients died in the non-ICU COVID department. 

During the same period, a total of 2570 BC sets were obtained from 1422 patients hospitalized in non-COVID-19 departments. Of them, 692 BC sets (26.9%) were detected as positive. True pathogens were recovered from 416 BC sets. Contamination rate was 39.9%. A total of 343 BSI episodes in 294 patients (male 48%, median age 77.5 years [IQR (25–75): 65–86]) were recorded among 19,973 patients hospitalized in non-COVID-19 departments (1.5%). The median time of hospitalization from admission to the onset of the BSI episode was 4 days [IQR (25–75): 0–21]. Nosocomial BSI accounted for 55.7% of all episodes.

**Figure 1:**
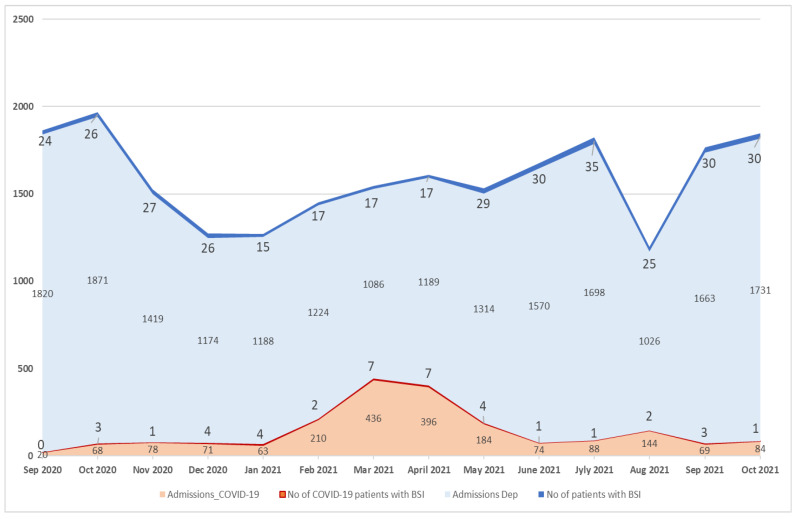
Monthly number of BSI episodes and monthly admissions in COVID-19 and non-COVID-19 departments, except ICU.

Monthly number of BSI episodes and monthly admissions in the COVID-19 department and all (except ICU) medical and surgical departments of the hospital are shown in Figure 1. The mean monthly incidence of BSI per 100 admissions was 2.015 (range: 0–6.349) in COVID-19 departments vs. 1.742 (range: 1.319–2.437) in non-COVID-19 departments. 

Blood isolates in COVID-19 and non-COVID-19 departments are presented in Table 1.

Of note, blood-culture contamination rate was lower in non-COVID-19 departments (39.9%) than in COVID-19 ones (52%). In the COVID-19 group, Gram-positive bacteria accounted for 46.4%, Gram-negative bacteria for 33.9% and fungi (candida isolates) for 19.6% of all pathogens. Gram-negatives prevailed in the non-COVID-19 group (59%). The rate of polymicrobial BSI did not differ between groups (24.4% vs. 20.7%). Candidemia and enterococcal bacteremia were significantly more frequent in COVID-19 than in non-COVID-19 patients (*p* = 0.018 and *p* < 0.001, respectively) (Table 1). Among *Candida* isolates, *Candida parapsilosis* was more frequently identified in the COVID-19 vs. the non-COVID-19 group (Supplementary Table S2). Candidemia was more often detected in patients with a CVC in place compared to patients without a CVC [*n* = 8 (44.4%) vs. *n* = 2 (9.1%), *p* = 0.025]. The outcome of COVID-19 patients with BSI did not significantly differ among pathogens (data in Supplementary Table S1).

### 3.2. Antimicrobial Resistance

**Figure 2:**
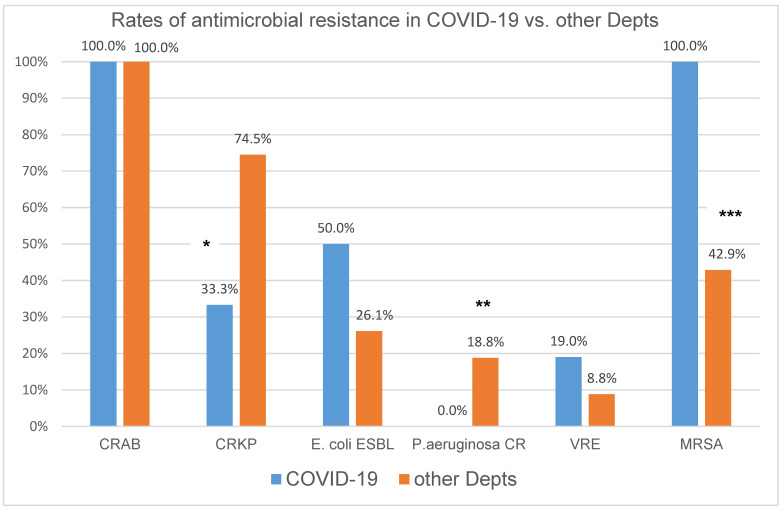
Resistance profiles of blood isolates among the COVID-19 department and all other non-ICU departments. Footnotes: CRAB—carbapenem resistant *Acinetobacter baumannii*; *CRKP*—carbapenem-resistant *Klebsiella pneumoniae*; *P. aeruginosa CR* (carbapenem-resistant); VRE—vancomycin resistant Enterococci; MRSA—methicillin-resistant Staphylococcus aureus; statistical significance * *p* = 0.047, ** *p* = 0.002, *** *p* = 0.042.

Resistance profiles of blood isolates among the COVID-19 department and all other non-ICU departments of the hospital are presented in Figure 2. Multidrug resistance was detected in 46% of pathogens in the COVID-19 group vs. 36.6% in the non-COVID-19 group (*p* = 0.769). All *A. baumannii* isolates were carbapenem-resistant in both groups and high rates of colistin resistance were noted (87% in the COVID-19 group and 77.1% in the non-COVID-19 group, *p* = 0.666). All *S. aureus* isolates were MRSA in the COVID-19 group vs. 42.9% in the non-COVID-19 cohort (*p* = 0.042). VRE accounted for 19% of enterococcal bacteremia in COVID-19 vs. 8.8% in the non-COVID-19 patients (*p* = 0.231). Half of *E. coli* isolates were ESBL producers in the COVID-19 group vs. 26.1% in the non-COVID-19 group (*p* = 0.3). Carbapenem-resistant *K. pneumonia* (CRKP) and *P. aeruginosa* predominated in the non-COVID-19 cohort vs. COVID-19 (74.5% vs. 33.3%, *p* = 0.047 and 18.8% vs. 0%, *p* = 0.002, respectively). The single carbapenemase-producing *K. pneumoniae* isolate detected in the COVID-19 group was a KPC and VIM co-producer. In the non-COVID-19 group, among all CRKP, KPC producers predominated (66.7%). 

### 3.3. Case-Control Analysis for Clinical Data and Outcome

Multiple comorbidities (i.e., heart disease, dyslipidemia and neuropsychiatric disorders), presence of CVC and prior hospitalization were significantly more frequent in the BSI group. Severe pneumonia and lack of vaccination against SARS-CoV-2 predominated in the BSI group. Patients with BSI were more frequently intubated (40% vs. 2.5% in the non-BSI group, *p* < 0.001) and had a longer length of hospital stay than non-BSI patients [mean days ±SD: 19.92 (9.56) vs. 9.57 (6.58), *p* = 0.001] (Table 2).

All cases of adverse outcome of COVID-19 patients—both in BSI and non-BSI group—were related to severe SARS-CoV-2 pneumonia [OR (95% CI): 2.238 (1.628–3.076), *p* < 0.001] and lack of vaccination [OR (95% CI): 0.606 (0.499–0.736), *p* = 0.003] (Table 3). In the multivariate-adjusted model including BSI, age > 60 years, and neurological and psychiatric disease, only BSI and neurological disease were associated with adverse outcome (Table 3). The same regression analysis including age >60 years, BSI and more than two of any comorbidity revealed BSI as the unique independent predictor of death/and or IMV during hospitalization [OR (95% CI): 21.47 (3.86–119.21), *p* < 0.001].

## 4. Discussion

We conducted a comparative analysis of microbial epidemiology and antimicrobial resistance profiles in BSI among COVID-19 and non-COVID-19 patients. Moreover, we analyzed the clinical characteristics and the outcome of patients with BSI and COVID-19 compared to non-BSI COVID-19 patients hospitalized in medical wards. The novelty of our study is a clear incorporation of a COVID-19 population outside the ICU environment. The incidence of BSI in COVID-19 patients and their outcome has been studied mostly in ICU or in mixed populations of both ICU and medical wards [11]. Furthermore, epidemiology of bacterial and fungal pathogens in BSI of patients with COVID-19 was mostly described in ICU departments with endemicity of multidrug antimicrobial resistance, but not in non-ICU COVID-19 settings [20].

In our cohort, the mean incidence of BSI (2.015 per 100 admissions) was lower than the reported rates in COVID-19 wards outside the ICU [11,21]. However, our results confirmed previous reports of higher BSI incidence in COVID-19 vs. non-COVID-19 patients [11]. 

The role of BSI contaminants in COVID-19 departments has been highlighted. The rate of blood-culture contamination in our study was high (52%), being higher than that observed in non-COVID-19 departments. High percentages of CoNS without evidence of increased catheter-related BSI (CRBSI), raise a higher suspicion of contamination and may result in additional unnecessary antimicrobial use. BSI contaminants were more frequently detected during the pandemic vs. the pre-pandemic era [22,23,24]. A double rate of BSI contaminants in COVID-19 vs. non-COVID-19 patients was also described [25]. During early hospitalization for COVID-19, the rates of bacterial and fungal co-infection are low, therefore overutilization of blood cultures is not justified [26,27]. During the COVID-19 waves, reduced compliance with blood sampling protocols and guidelines for the placement and management of CVCs have been observed [23,24,28]. Moreover, antimicrobial stewardship is required as empirical antimicrobial treatment in patients with COVID-19 for unjustified diagnosis of bacterial co-infection is high, raising concerns regarding the emergence of antimicrobial resistance [28,29,30].

As epidemiology of BSI in COVID-19 patients, similar to non-COVID-19 patients, varies among countries and continents, continuous surveillance is required [12]. Our study demonstrated high rates of MDR pathogens, as also reported from another hospital in our country [20]; both studies mirror the endemic spread of MDRO, especially Gram-negatives. Protonotariou et al. reported a higher rate of MDRO (58% vs. 46% in our study) possibly because ICU patients prevailed in that cohort. In our study, all *A. baumannii* isolates detected in both COVID-19 and non-COVID-19 BSI episodes were carbapenem-resistant, suggesting a nosocomial horizontal spread throughout hospital departments. *A. baumannii* outbreaks during the initial surge of COVID-19 could be partly explained by the shortages in personnel, personal protective equipment (PPE) and dedicated medical equipment, which had affected the conventional infection prevention and control (IPC) practices [31]. MDR *A. baumannii* was the main BSI pathogen in a COVID-19 ICU (44%) in Spain [32]. The increased prevalence of *A. baumannii* either as a colonizer or as a pathogen during the COVID-19 pandemic has not been fully explored. In our cohort, colonization screening among COVID-19 patients was not routinely performed. Contrary to *A. baumannii*, other carbapenem-resistant Gram-negatives, such as *K. pneumoniae* and *P. aeruginosa*, were more frequently found in the non-ICU non-COVID-19 wards. Current published data suggest that bacteremia caused by *K. pneumoniae* and *A. baumannii* are mainly ICU-acquired in COVID-19 patients [14]. 

MRSA bacteremia in COVID-19 patients was mostly detected upon admission [14,33]. In our study, all *S. aureus* isolates were MRSA, though detected in only four cases; these episodes did not clearly originate from the healthcare environment and had no impact on mortality. On the contrary, mortality of *S. aureus* bacteremia during the COVID-19 pandemic was reported to be high, over 60% in larger studies [34,35]. 

Enterococci are a major cause of BSI in COVID-19 patients hospitalized in ICUs. This may be due to enteric involvement in patients with severe disease, colonization pressure by antibiotics and/or limitations in controlling the patient-to-patient transmission in extremely challenging circumstances [36]. However, molecular analysis of *Enterococcus* spp. clinical isolates from COVID-19 patients did not always confirm a horizontal nosocomial transmission [37]. In our study, enterococcal bacteremia was significantly more frequent in COVID-19 patients than in non-COVID-19 patients with a high rate of VRE. We have no data on genetic relatedness of the isolates. It is presumed that prior antibiotic consumption in the community by outpatient physicians or over the counter, can alter gut flora and facilitate VRE colonization and subsequent infection. 

Candidemia is increasingly reported in COVID-19 patients usually associated with ICU stay, need for IMV, use of corticosteroids or other immunosuppressive drugs for COVID-19 [38,39]. Mortality has been reported to be higher in patients with candidemia and COVID-19 than in patients without COVID-19 [39]. Our study corroborates the higher incidence in COVID-19 vs. non-COVID-19 patients in non-ICU wards, demonstrating that other factors might interact with the host during the course of the disease, allowing invasive candidiasis. The alterations of microbiota from enteritis of COVID-19 or/and the prior use of antimicrobials and subsequent gut or mucosal colonization by *Candida* spp. might be implicated in the pathogenesis of candidemia [39]. 

High mortality rates (57%) have been reported among COVID-19 patients with BSI following an increased need for IMV (74%) [21]. Several risk factors for the development of BSI have been described in COVID-19 patients, similarly to influenza infection cohorts [40]. Bacteremia has been strongly correlated with ICU stay, the presence of an indwelling device, underlying comorbidities and adverse clinical outcome [41]. In a large cohort of 13,007 patients originally recruited followed by a propensity-score-matching cohort of 6520 patients, 3.74% and a 3.97% were diagnosed with clinically significant BSI, respectively. COVID-19 patients with BSI had significantly longer hospitalizations, more frequent ICU admission and higher mortality rates compared to those without BSI [42]. In our study, lack of vaccination for SARS-COV2 was not only correlated to severe pneumonia but also to an increased frequency of BSI, further hampering the patients’ outcome. As already published [43], BSI in hospitalized patients with COVID-19 has the most significant impact on mortality.

Standards of care during the COVID-19 pandemic seemed to be altered, shifting to a less frequent and per protocol oral care and/or washing/dressing and CVC management, further complicated by the prone position of the patient. In addition, the rapidly increasing burden of workload and the pressure on healthcare professionals has a negative impact on infection-control practices [44]. Therefore, during the first wave of COVID-19, the rates of CRBSI had increased in a Dutch hospital but dropped again to less than 9% after implementation of infection-control measures. The decrease in CRBSI rates following the adaptation of the infection-control policies, to meet the needs of the new hospital environment for COVID-19, proves that the key to avoid life-threatening BSI episodes is the application of feasible and effective infection-control interventions [23]. This is only an example of how surveillance data on nosocomial infections can improve actions for infection control. Maintaining IPC best practices (e.g., MDRO surveillance, hand hygiene, standard and contact precautions and environmental cleaning) could mitigate the spread of infections [31]. 

## 5. Conclusions

In conclusion, our cohort demonstrated a relatively high incidence of MDRO in BSI complicating COVID-19 infection in patients hospitalized in medical wards outside the ICU environment. Neither cross-transmission nor gut pre-colonization—especially for VRE, ESLB-producing *E. coli* and *Candida* spp.—as a portal of the pathogen can be excluded. A limitation of our study is the lack of information on colonization rates and molecular analysis for clusters of BSI isolates. Additionally, our study is a single-center study in a highly endemic area for MDRO; therefore, our results cannot be generalized. However, via a case-control analysis, we highlighted the conditions that correlate with BSI in COVID-19 patients in medical wards. We confirmed that BSI is the strongest predictor of IMV and in-hospital mortality in a non-ICU population of patients with COVID-19, considered to be less prone to severe complications and adverse outcome. 

The COVID-19 pandemic revealed in a dramatic way the urgent necessity for implementation of efficacious infection-control policies with a bundle of interventions in order to reduce BSI and other co-infection in COVID-19 units. Compliance with guidelines for infection control and standards of care are imperative [15,45].

## Figures and Tables

**Table 1:** Blood isolates in COVID-19 and non-COVID-19 departments. Bold means statistical significant values.

	Non-COVID-19 Departments ^1^		COVID-19 Department ^1^		
Pathogens	*n*	%	*n*	%	*p*
*Acinetobacter baumannii* complex	35	8.16%	8	14.3%	*0.135*
Anaerobes	9	2.10%			
*Candida* sp.	38	**8.86%**	11	**19.64%**	**0.018**
*Enterobacter cloacae* complex	5	1.17%			
*Enterococcus* spp.	59 ^2^	**13.75%**	21 ^3^	**37.5%**	**<0.001**
*Escherichia coli*	69	16.08%	4	7.1%	0.109
*Klebsiella* sp. (other than pneumoniae)	6	1.40%			
*Klebsiella pneumoniae*	55	12.82%	3	5.4%	0.126
Other Gram-negatives	21	4.90%	1	1.8%	
Other Gram-positives	4	0.93%			
*Proteus mirabilis*	18	4.20%	1	1.8%	0.711
*Pseudomonas aeruginosa*	32	7.46%	1	1.8%	0.157
*Serratia marcescens*	5	1.17%			
*Staphylococcus aureus*	56	13.05%	4	7.1%	0.280
*Stenotrophomonas maltophilia*	7	1.63%	1	1.8%	>0.999
*Streptococcus* sp.	10	2.33%	1	1.8%	>0.999
**Total**	**429**		**56 ^4^**		

Footnotes: ^1^ Except for ICU department; ^2^ *E. faecalis*, *n* = 37, *E. faecium*, *n* = 20, *E. casseliflavus*, *n* = 1, *E. gallinarum*, *n* = 1; ^3^ *E. faecalis*, *n* = 7, *E. faecium*, *n* = 14; ^4^ In 11/45 episodes two pathogens were detected.

**Table 2:** Case-control analysis of clinical characteristics of patients with COVID-19 between BSI and non-BSI groups. Bold means statistical significant values.

Variables	Total, *n* = 80	BSI Group *n* = 40	Non-BSI Group *n* = 40	*p*
Male *n* (%)	38 (47.5)	19 (47.5)	19 (47.5)	>0.999
Female *n* (%)	42 (52.5)	21 (52.5)	21 (52.5)	
Age, mean (±SD)	71.14 (14.28)	71.20 (14.25)	71.08 (14.48)	0.923
Prior hospitalization *n* (%)	15 (18.8)	13 (32.5)	2 (5)	0.003
Vaccination for COVID-19 *n* (%)	14 (17.5)	1 (2.5)	13 (32.5)	0.001
Comorbidities				
Heart disease *n* (%)	28(35)	20 (50)	8 (20)	0.009
Hypertention *n* (%)	47 (58.8)	26 (65)	21 (52.5)	0.364
Stroke *n* (%)	8 (10)	6 (15)	2 (5)	0.263
Peripheral vascular disease *n* (%)	16 (20)	15 (37.5)	1 (2.5)	<0.001
Diabetes mellitus	28 (35)	17 (42.5)	11 (27.5)	0.241
Dyslipidemia *n* (%)	34 (42.5)	23 (57.5)	11 (27.5)	0.012
Respiratory disease *n* (%)	15 (18.8)	12 (30)	3 (7.5)	0.020
Chronic kidney disease *n* (%)	10 (12.5)	5 (12.5)	5 (12.5)	>0.999
Neurological disease *n* (%)	15 (18.8)	12 (30)	3 (7.5)	0.020
Psychiatric conditions *n* (%)	13 (16.5)	13 (32.5)	0	<0.001
Malignancy *n* (%)	9 (11.3)	6 (15)	3 (7.5)	0.481
Autoimmune diseases	10 (12.5)	7 (17.5)	3 (7.5)	0.311
More than 2 comorbidities	41 (51.2)	31 (77.5)	10 (25)	<0.001
Severe SARS-CoV2 pneumonia *n* (%)	47 (58.8)	33 (82.5)	14 (35)	<0.001
Central venous catheter *n* (%)	25 (31.3)	18 (45)	7 (17.5)	0.015
Length of hospital stay mean (±SD)	14.68 (9.66)	19.92 (9.56)	9.57(6.58)	0.001
Need for mechanical invasive ventilation–ICU transfer *n* (%)	17 (21.3)	16 (40)	1 (2.5)	<0.001

**Table 3:** Analysis of factors related to adverse outcome of COVID-19 patients in medical wards.

Variables	Discharge Home *n* = 54 (67.5%)	Adverse Outcome *n* = 26 (32.5%)	Univariate OR (CI 95%)	*p*	Multivariate Adjusted OR (CI 95%)	*p*
Age > 60 years	42 (77.8)	22 (84.6)	1.571 (0.453–5.450	0.562	1.345 (0.276–6.564)	0.714
Male Female	24 (44.4) 30 (55.6)	14 (53.8) 12 (46.2)	1458 (0.570–3.731)	0.480		
Vaccination for COVID-19	14 (28.9)	0 (0)	0.606 (0.499–0.736)	0.003		
Prior hospitalization-transfer from hospital/nursing care	7 (13)	8 (30.8)	2.984 (0.941–9.431)	0.071		
Comorbidities						
History of Stroke	3 (5.6)	5 (19.2)	4.048 (0.886–18.486)	0.105		
Heart Disease	16 (29.6)	12 (46.2)	2.036 (0.774–5.356)	0.211		
Arterial hypertension	30 (55.6)	17 (65.4)	1.511 (0.573–3.986)	0.472		
Dyslipidemia	20 (37)	14 (53.8)	1.983 (0.768–5.120)	0.227		
Respiratory disease	11 (20.4)	4 (15.4)	0.711 (0.203–2.492)	0.763		
Diabetes mellitus	17 (31.5)	11 (42.3)	1.596 (0.607–4.198)	0.453		
Malignancy	5 (9.3)	4 (15.4)	1.782 (0.436–7.282)	0.462		
Renal failure	6 (11.1)	4 (15.4)	1.455 (0.373–5.679)	0.720		
Peripheral vascular disease	8 (14.8)	8 (30.8)	2.556 (0.833–7.843)	0.135		
Autoimmune diseases	6 (11.1)	4 (15.4)	1.455 (0.373–5.679)	0.720		
Psychiatric disorders	3 (5.6)	10 (38.5)	10.625 (2.601–43/395)	<0.001	2.325 (0.476–11.369)	0.297
Neurological disorders	4 (7.4)	11 (42.3)	9.167 (2.545–33.022)	<0.001	**5.704** **(1.103–29.495)**	**0.038**
Bacteremia	16 (29.6)	24 (92.3)	28.500 (6.011–135.121)	<0.001	**19.512** **(3.672–103.668)**	**<0.001**
Severe SARS-CoV-2 pneumonia	21 (38.9)	26 (100)	2.238 (1.628–3.076)	<0.001		

## Data Availability

Not applicable.

## Supplementary Materials

The following supporting information can be downloaded at: https://www.mdpi.com/article/10.3390/microorganisms10071314/s1, Supplementary Table S1: Identification of *Candida* spp.; Table S2: Pathogen-related in-hospital outcome in patients with BSI.
