# Supplementary material for: Bloodstream Infections in a COVID-19 Non-ICU Department: Microbial Epidemiology, Resistance Profiles and Comparative Analysis of Risk Factors and Patients’ Outcome
Source: Microorganisms. 2022 Jun 29;10(7):1314. doi: 10.3390/microorganisms10071314 (PMC9318208; doi:10.3390/microorganisms10071314)
Supplement: Supplementary file 1 [file microorganisms-10-01314-s001.zip › microorganisms-1727987-supplementary.pdf]

Supplementary tables

Table S1. Identification of *Candida* spp.

Candida spp	**Non-COVID-19 patients**		**COVID-19 patients**	
	N	%	N	%
Candida albicans	17	44.7	5	45.45
Candida auris	2	5.3		
Candida dubliniensis	1	2.6		
Candida glabrata	3	7.9		
Candida lusitaniae	2	5.3		
Candida parapsilosis	12	31.6	5	45.45
Candida tropicalis	1	2.6		
Candida palmioleophila			1	9.1
Total	38		11	

Table S2. Pathogen-related in-hospital outcome in patients with BSI.

**Pathogens**	**Cure,n=16 (40%)**	**no-Cure,n=24 (60%)**	***p***
Staphylococcus aureus	2(12.5)	2(8.3)	>0.999
Enterococcus spp[1]	9(56.3)	11(45.8)	0.748
GRAM negative bacteria	5(31.3)	9(37.5)	0.746
Candida spp	2(12.5)	8(33.3)	0.263

Footnotes

[1] Including Enterococcus faecalis and Enterococcus faecium

[2] Including Acinetobacter baumannii, Klebsiella pneumoniae, Escherichia coli, Pseudomonas aeruginosa, Proteus mirabilis, Stenotrophomonas maltophilia, Bordetella bronchiseptica

[3] Including Candida albicans, Candida parapsilosis, Candida palmioleophila
